# To what extent should doctors communicate diagnostic uncertainty with their patients? An empirical ethics vignette study

**DOI:** 10.1136/jme-2024-109932

**Published:** 2025-02-26

**Authors:** Caitríona Cox, Thea Hatfield, Matthew Parry, Zoë Fritz

**Affiliations:** 1 The Healthcare Improvement Studies Institute, The University of Cambridge, Cambridge, UK; 2 Department of Mathematics and Statistics, University of Otago, Dunedin, New Zealand

**Keywords:** Ethics, Decision Making, Ethics- Medical

## Abstract

**Background/aims:**

Although diagnostic uncertainty is common, patient-focused research examining its communication is lacking. We aimed to determine patient preferences for the communication of diagnostic uncertainty, and examine the effects of such communication on patients.

**Methods:**

We applied an empirical ethics approach, integrating the data collected with ethical analysis to form normative recommendations about diagnostic uncertainty communication. In this randomised crossover study, n=111 members of the public sequentially watched two video vignettes depicting either high or low communicated diagnostic uncertainty, in one of two clinical scenarios. After watching videos, participants completed online questionnaires. Primary outcome was preferred video (high vs low communicated uncertainty); secondary outcomes included satisfaction, trust, worry and understanding. Quantitative data were analysed using logistic regression and a linear mixed effects model; qualitative data were analysed thematically.

**Results:**

Quantitative analysis demonstrated that participants preferred greater diagnostic uncertainty communication, even though these vignettes were more worrying. Qualitative data revealed heterogeneous participant views justifying their communication preferences. These data raise issues relating to how doctors might balance harms versus benefits in diagnostic uncertainty communication and how doctors might communicate in the face of heterogeneous patient information preferences.

**Conclusions:**

We argue that doctors should err on the side of greater diagnostic uncertainty communication: to not do so (eg, based on benign paternalistic ideas about avoiding patient worry) or to do so variably (eg, based on unevidenced assumptions about patient information preferences) risks depriving patients of information they may value and may create or exacerbate inequalities.

## Introduction

### Ethical approaches to non-disclosure in the doctor-patient relationship

 Under what circumstances it is acceptable—ethically and legally—for doctors to *not* disclose information to their patients has been debated in various scenarios: regarding prognosis,^1^,^3^ risks of treatment^4^ and medical errors,^5^ to name but a few.

Historically, doctors took a relatively paternalistic approach to doctor-patient communication: it was considered acceptable, for example, to withhold information such as a diagnosis of terminal cancer to avoid distress.^6^ Over the latter half of the twentieth century, there has been a gradual shift (particularly in Western medicine) towards a less paternalistic conceptualisation of the doctor-patient relationship,^7^ with emphasis on open communication to support shared decision-making.^8 9^ This shift has also been reflected in case law, which has scrutinised standards of disclosure: in the UK, *Montgomery vs Lanarkshire*^10^ examined the duty of the doctor to disclose information about material risks of a given treatment, and any reasonable alternative treatments.

Despite these changes, there is still recognition that it is acceptable for doctors to withhold some information from patients. As Jones summarises, various justifications have been proposed:

*Patients will worry, and become too anxious about their treatment if they are given full information; they will not understand the information in any event, because it is too complex and technical…doctors do not have time to give full information to patients-there are too many patients and it would take too long*…^11^

Much of the ethical literature exploring medical non-disclosures employs a broad harm-benefit analysis: in determining the acceptability of a non-disclosure, there is consideration of its potential impact (both on the patient and healthcare system).^4 12 13^ Many analyses use Beauchamp and Childress’ ‘Four Pillars’^14^ as a scaffold, framing the fundamental ethical tension as arising from the need to balance competing ethical principles—particularly respect for autonomy (which would often favour greater disclosure of information) versus non-maleficence (which may favour non-disclosure of information which is distressing, overwhelming or unwanted).^15 16^

Such ethical arguments often rely on claims about the *effects* of communication on patients. It is thus important to understand them; in the absence of such understanding, incorrect assumptions about what information patients want, or how they will respond to it, may result in flawed conclusions about the ‘right’ approach. An example of this can be seen in palliative care: although historically communication about poor prognosis was limited by the perception that patients did not want or would be harmed by such information, empirical research has repeatedly demonstrated that patients value honest communication of prognosis (even if the prognosis is poor).^1 2 17 18^

### Communicating diagnostic uncertainty

One area of ongoing debate relates to communication about diagnosis.^19^ Diagnosis is not a single event, but a complex and collaborative process, which often involves considerable uncertainty.^20^ This uncertainty represents a communication challenge: to what extent, if at all, should doctors disclose diagnostic uncertainty to their patients?

Both researchers^20^,^22^ and regulatory bodies^23^ have made recommendations that doctors should communicate diagnostic uncertainty: for example, UK General Medical Council guidance states that ‘[i]f you are uncertain about the diagnosis… you should explain this to the patient’.^23^ There is, however, evidence that diagnostic uncertainty is *not* always communicated. Our recent study, which used written vignettes to explore doctor communication, found significant variation in practice: doctors did not always explicitly disclose diagnostic uncertainty, even when they were not themselves certain about the diagnosis.^24^

**Figure 1:**
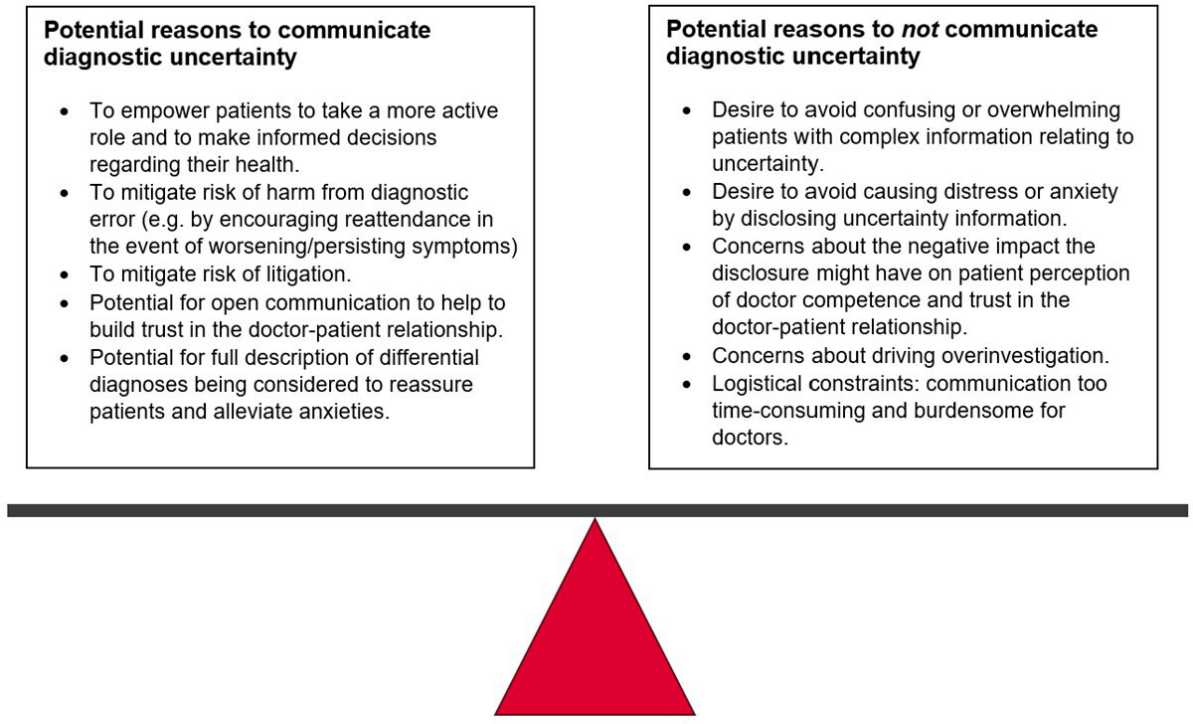
Considerations identified in existing literature about whether doctors should communicate diagnostic uncertainty to their patients.

That vignette study,^24^ and two systematic reviews,^25 26^ explored reasons why doctors choose to (not) disclose diagnostic uncertainty to patients. As outlined in figure 1, we identified a range of considerations. Some of these (eg, the desire to avoid patient worry) have been used to justify both the disclosure *and* the non-disclosure of diagnostic uncertainty. Communication of uncertainty could plausibly increase patient worry (by acknowledging the possibility of unlikely but serious differential diagnoses) *or* decrease it (by explicitly addressing concerns patients might already have about such diagnoses). Such opposing ideas might explain some of the observed variation in communication: although doctors often have overlapping communication *goals* (such as reducing patient worry), different ideas about how to achieve these goals result in differing communication *behaviours*.

A lack of patient-focused research in this area means that there is little empirical evidence to confirm or refute beliefs about the consequences of diagnostic uncertainty communication.^25 26^ While some evidence suggests that communicating uncertainty regarding treatment options may be associated with lower patient satisfaction,^27^ little research has directly examined the effects of communicating *diagnostic* uncertainty. One study in paediatrics found that explicit expression of diagnostic uncertainty was associated with lower perceived competence, less trust and confidence,^28^ while another study involving patients with endometriosis demonstrated a patient preference for clear communication of diagnostic uncertainty to facilitate informed decision-making.^29^

Questions also remain regarding the role of communicating diagnostic uncertainty when ‘safety-netting’ (giving advice to patients to help them identify when to seek further medical help if their symptoms worsen/do not improve).^30^ Some have suggested that sharing uncertainty is an important component of effective safety-netting,^31^,^34^ and there is evidence that some doctors explicitly share diagnostic uncertainty to encourage appropriate attendance and protect against diagnostic errors.^24 35^ However, whether communicating diagnostic uncertainty actually makes safety-netting more effective in encouraging patients to reattend in the event of worsening/non-resolving symptoms remains unclear.

### What does the current study add?

In summary, doctors have differing opinions about whether communicating diagnostic uncertainty to patients causes harm (eg, by causing worry or by overwhelming patients with complex details) or benefit (eg, by encouraging appropriate reattendance through effective safety-netting or by fostering a more trusting doctor-patient relationship). A dearth of relevant patient-focused research makes it difficult to establish which assumptions about the impact of diagnostic uncertainty communication are correct. As ethical analyses often rely on such assumptions, the lack of empirical evidence is problematic.

This study begins to address this research gap. We aimed to (1) determine patient preferences for the communication of diagnostic uncertainty and (2) examine the effects of communicating diagnostic uncertainty on patients. By exploring the impact of communicating high or low diagnostic uncertainty on a range of outcomes, this study helps to address the normative question of how doctors should communicate diagnostic uncertainty to their patients.

## Methods

### Overarching empirical ethics approach

Empirical ethics refers broadly to research which combines the use of empirical data with moral theory to reach normative conclusions.^36^,^39^ A wide range of methodologies exist within this growing field.^36^ These vary regarding their integration of the empirical and theoretical—different approaches use different ‘bridging methodologies’ to combine empirical and conceptual analysis to produce normative solutions to identified ethical problems.^38^

The study presented here forms part of a wider programme of research, which uses an empirical ethics approach to explore issues relating to the formation, communication and recording of diagnoses. This strand of the project has examined the communication of diagnostic uncertainty, ultimately addressing the normative question of to what extent doctors should communicate diagnostic uncertainty to their patients.

We applied Huxtable and Ives’ ‘Mapping-Framing-Shaping’ framework.^38^ Two systematic reviews helped to ‘map’ the area, identifying what was known and what remained unexplored regarding the communication of diagnostic uncertainty and identifying associated ethical issues.^25 26^ We then used vignette methodology to ‘frame’ relevant topics, collecting empirical data to explore: (1) how and why doctors currently communicate diagnostic uncertainty, (2) what the impact of such communication is on patients and (3) what patient preferences for such communication are. We finally aim to ‘shape’ the terrain by using wide reflective equilibrium^40^ to synthesise the empirical data with moral theories regarding information disclosure within the doctor-patient relationship, to suggest normative recommendations about how doctors should approach the communication of diagnostic uncertainty.

In this paper, we present the empirical data from the patient-focused video vignette study. We discuss the results with reference to ethical theory and the existing literature, referring to work exploring therapeutic privilege and ethical aspects of non-disclosure within the doctor-patient relationship.

### Study design

In observational studies of real consultations, specific communication behaviours cannot be isolated and manipulated: they can only explore correlations between communication behaviours and outcomes measures. In contrast, our use of vignette methodology in this study allowed controlled manipulation of the degree of diagnostic uncertainty communication (permitting consideration of causal relationships between uncertainty communication and relevant outcomes).

**Figure 2:**
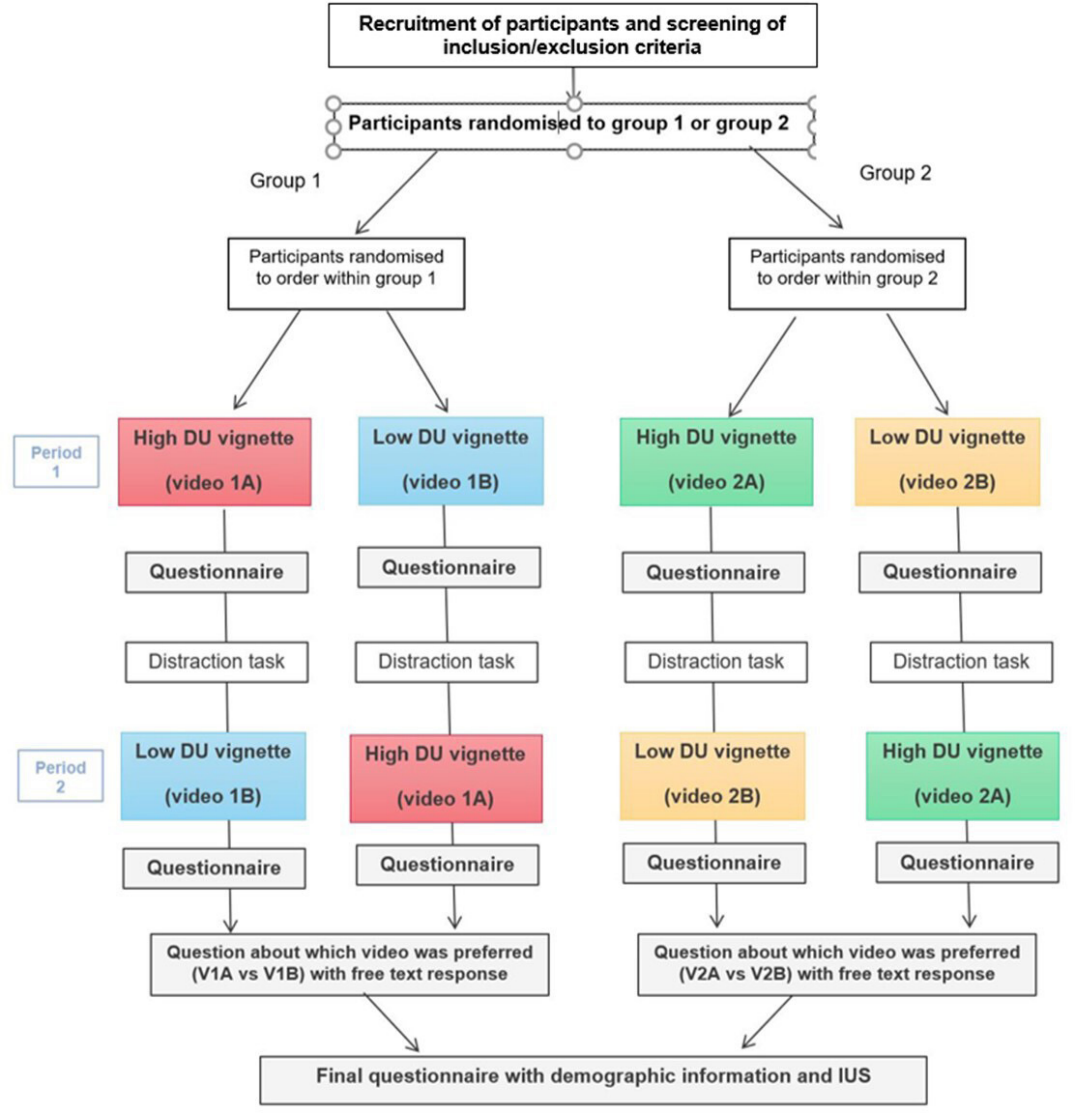
Study design.DU, diagnostic uncertainty; IUS, intolerance to uncertainty scale.

This was a randomised crossover study. Participants sequentially watched two video vignettes depicting either high or low communicated diagnostic uncertainty. We used two separate clinical scenarios: participants were initially randomised to either group 1 (headache), or group 2 (change in bowel habit (CoBH)) and then were further randomised within group to the order of vignettes watched (figure 2).

We administered the vignettes using Thiscovery, an online platform developed by THIS institute. Participants engaged with the study alone using their own electronic devices, without researcher supervision. Videos could not be paused or rewatched.

### Development of vignettes

The process of developing the vignettes, including pilot-testing, is reported elsewhere.^41^ In brief, we followed published guidance on the development of video vignette studies^42^ to produce two versions (high vs low communicated uncertainty) of two clinical scenarios. We drew on data from an earlier vignette study involving doctors to make the scripts as realistic as possible.^24^ The high communicated uncertainty vignettes included a range of verbally expressed implicit and explicit statements of uncertainty; the low communicated uncertainty vignettes did not include acknowledgement of diagnostic uncertainty. Other verbal and non-verbal elements of the consultation were kept constant between the vignettes.

### Outcome measures

Participants completed questionnaires: (i) after watching each vignette individually; (ii) after watching both (asking which video was preferred, including an optional free text space to explain this preference); and (iii) to record demographic information, previous personal experience of the clinical scenario and a 12-item intolerance to uncertainty scale (IUS-12).^43^ We chose to ask participants to complete the IUS *after* watching the vignettes rather than before, to avoid priming them think about uncertainty.

The primary outcome measure was preference regarding diagnostic uncertainty communication (ie, preference for video A or B, determined after watching both vignettes).

Secondary outcome measures were identified via a combination of literature review, the results of a previous study conducted by the research team^24^ and patient public interest (PPI) group discussion. They included:

1. Patient overall satisfaction with the consultation.
2. Patient worry.
3. Patient perception of doctor competence.
4. Patient trust in the doctor.
5. Patient understanding of information provided.
6. Patient happiness with the amount of information provided.
7. Patient desire for further investigations.
8. Patient likelihood of seeking further medical attention if their symptoms persisted/worsened

Secondary outcomes were assessed using single-item 0–10 numerical rating scales. We included verbal anchors at the extremes and midpoint to improve the reliability and validity.^44 45^ We used various sources to develop the items (including literature review and consultation with proposed respondents) to increase face validity.^46^

The questionnaires can be found in online supplemental appendix A, alongside a referenced explanation for our decision to use single-item measures as opposed to multi-item scales.

### Participants, power and recruitment

Participants were members of the UK public, aged 18 years and above. They all provided informed consent via an online form. Medical students and doctors were excluded. There were two separate recruitment approaches:

1. General recruitment: advertisement via social media and cascading via individuals/networks.
2. Partner organisations: four Healthwatch (an independent national champion for people who use health/social care services) organisations in different locations were contracted to each recruit ten participants from demographics that were proving harder to reach via social media recruitment (eg, non-university educated, those at extremes of age).

In performing the power calculations, we assumed a significance level of 0.05, a minimum detectable difference in means of 1 and a within subject SD of 1. For 90% power, we would need a minimum of 48 subjects in total (ie, 12 in each group). We planned to recruit more than this, aiming for n=100 (ie, 25 subjects in each group), to capture a wider range of population characteristics.

### Data analysis

We used a mixed methods approach: the final questionnaire combined quantitative and qualitative data (free text responses qualifying reasons for vignette preference). These data were integrated at the interpretation stage of the study and were given equal priority.^47 48^ Analysis of embedded qualitative responses was intended to augment and explain complex or contradictory quantitative responses.^49^ By combining statistics with thematic analysis, we hoped to avoid over-reliance on the former, better capturing experiences and subjective factors necessary to elucidate complex phenommena.^48^

#### Quantitative analysis

For the primary outcome, logistic regression was used to estimate the proportion preferring vignette A (the video communicating high uncertainty), allowing for a potential scenario effect (headache or CoBH) and a potential sequence effect (AB or BA). A goodness-of-fit test (deviance test) was used to assess model adequacy. Adjustment was also made for IUS score and subject history. In the analysis of the primary outcome, a significance level of 0.05 was chosen.

Each secondary outcome was treated as a continuous response and a linear mixed effects model was used to estimate the (fixed) effect of vignette type, allowing for a possible ‘carry-over’ effect.^1^ The random effect allowed for between-subject variability but was constant for within-subject responses. The advantage of using a linear mixed effects model over a simpler model of within-subject differences is that adjustment can be made for IUS score and subject history of the condition. Graphical checks of the normality assumption for both random effects and residual errors were used to assess model adequacy. In the analysis of the secondary outcomes, a stringent significance level of 0.01 was chosen, to mitigate the effect of multiple testing leading to false discoveries (Type I errors).

To determine whether individuals had been appropriately randomised into groups of scenario and sequence, Fisher’s exact test was used on the demographic data and a t-test was used on the IUS scores, with a significance level of 0.05.

Microsoft Excel and R^50^ were used.

#### Qualitative analysis

Qualitative data were analysed using reflexive thematic analysis.^51^,^53^ This is a theoretically flexible analytical approach, in which codes represent researcher interpretations of patterns of meaning across the dataset.^53^ Our team consisted of clinical and non-clinical researchers; we used this analysis approach with the aim of both acknowledging and embracing the reflexive influence of varying researcher interpretations grounded in different backgrounds/perspectives. Using an iterative, open coding approach, we developed codes to capture the main themes interpreted from the data. TH and CC initially familiarised themselves with the data, before independently developing codes. The whole research team then met to review these codes and determine the ongoing coding approach. CC then coded the entirety of the data using this approach. Higher-level themes were identified and were compared with existing literature to highlight significant trends and gaps. NVivo 12 Pro software was used.

## Empirical results

Data were collected in a continuous period from December 2022 to March 2023 (n=111 in total; table 1).

**Table 1:** Participant details

Participant characteristic	N (%)
Age	
18–29	13 (11.7)
30–39	12 (10.8)
40–49	12 (10.8)
50–59	24 (21.6)
60–69	16 (14.4)
70–79	28 (25.2)
80 or over	6 (6.3)
Sex	
Female	74 (66.6)
Male	36 (32.4)
Prefer not to say	1 (0.9)
Ethnicity	
English, Welsh, Scottish, NI or British	86 (77.4)
Other White background	5 (4.5)
Mixed/multiple ethnic groups	2 (1.8)
Asian/Asian British	9 (8.1)
Black/African/Caribbean	7 (6.3)
Arab	0 (0)
Other	1 (0.9)
Prefer not to say	1 (0.9)
Educational level	
Primary level	1 (0.9)
Secondary school (up to 16)	6 (5.4)
Higher, secondary or further education	19 (17.1)
University or college	53 (47.7)
Postgraduate degree	32 (28.8)
Region	
North East	0 (0)
North West	13 (11.7)
Yorkshire and the Humber	19 (17.1)
East Midlands	1 (0.9)
West Midlands	1 (0.9)
East of England	12 (10.8)
London	22 (19.8)
South West	16 (14.4)
Wales	1 (0.9)
Scotland	1 (0.9)
Northern Ireland	0 (0)
South East	28 (25.2)

NI, Northern Ireland.

### Quantitative results

The demographic data show the participants are representative of the UK population in terms of ethnicity. However, the study group is skewed in terms of age (67.5% are over the age of 50), sex (66.6% identified as female) and education (76.5% have a university or college degree, or higher). In terms of demographic characteristics and IUS score, participants were found to be appropriately randomised into groups scenario and sequence.

The expressed preferences by scenario and sequence are shown in table 2. The estimated proportion (of the population) preferring videos with high communicated diagnostic uncertainty is 0.64 with a 95% CI (0.55, 0.73). There is no evidence for a difference in the order the videos were viewed, and, there is no evidence that IUS-12 score of previous history of the clinical scenario has an effect on preference. There is no evidence for a difference in preference based on education level.

**Table 2:** Number of participants preferring each vignette (presented according to sequence watched)

Sequence	Preference A (high DU vignette)	Preference B (low DU vignette)
Headache scenario		
**AB**	20	4
**BA**	19	14
Change in bowel habit scenario		
**AB**	20	12
**BA**	12	10

DU, Diagnostic Uncertainty.

Analysis of the secondary outcomes (table 3) indicates statistically significant results for levels of worry. The estimated mean change in worry from viewing videos with high communicated diagnostic uncertainty is an increase of 1.14 on the 10-point scale with a 95% CI (0.60, 1.67). There is no evidence for a difference between the vignettes (headache vs CoBH scenarios). The results are also robust if adjustment is made for IUS-12, personal history of the medical condition and education (university or college degree or higher; or not).

**Table 3:** Effect of high communicated diagnostic uncertainty (video A) on the secondary outcomes (using a significance level of 0.01 as the threshold)

Secondary outcome	Estimate	99% CI
Satisfaction	0.21	(−0.35, 0.78)
Worry	1.14	(0.42, 1.85)
Perception of doctor competence	0.35	(−0.06, 0.77)
Trust in the doctor	0.17	(−0.30, 0.65)
Understanding of information provided	NA	NA
Desire for further investigations	0.31	(−0.70, 1.31)
Likelihood of seeking further medical attention in the event of worsening or non-resolving symptoms	0.31	(−0.23, 0.84)

Of note, the level of understanding could not be analysed as normality assumptions were violated; this was most likely because the distribution of responses was heavily skewed: 76% of responses were 9 or 10. This indicates that all versions of the vignettes produced (both high and low communicated diagnostic uncertainty) were highly understandable for participants.

### Qualitative results

After watching both high and low communicated diagnostic uncertainty vignettes, participants indicated their preference and were asked to provide reasons for this. These data revealed varied justifications. Common themes included: feelings about the detail provided, the perception of the communication as worrying versus reassuring, the impact of the communication on perceptions of the doctor and the impact of the communication on health behaviours.

As there was little difference between the responses given by participants who watched the headache vignettes versus the CoBH vignettes, we do not differentiate the data and present the main themes below.

#### Amount of information provided: desire for information versus overwhelming/unnecessary detail

The most common reason participants gave for preferring the high communicated uncertainty vignettes was a desire to be better informed. Many responded in general terms:

*I like to know as much as possible when asking for advice about anything from medical matters to making decisions about plumbing*. – P10

Some elaborated that greater information was helpful in facilitating better understanding of the situation; others explained their preference with specific reference to the discussion about the differential diagnosis or the rationale behind investigations. Understanding the doctor’s thought processes—including the handling of uncertainty and the differential diagnosis—was helpful.

*More relevant information was given and I felt I had a clearer picture of the situation*. – P1

*I like the thought process taken to come out with diagnosis*. – P94

This desire for more detail was not, however, universal: a minority felt that the amount of information provided in the high communicated uncertainty vignette was unnecessary.

*The [high communicated uncertainty video] included information I did not need, may not understand, created anxiety and expressed uncertainty about the quality of the investigation, diagnosis and treatment*. – P30

A few participants stated that they felt the level of detail provided should be patient-led—so if a patient wanted more information, they could ask for it.

*If I wanted more information about what they were specifically looking for, then I would ask.* – P60

#### Worry versus reassurance

A prominent theme was that of worry versus reassurance: participants frequently referred to ideas about the communication causing or relieving worry (see table 4 for illustrative quotations). Importantly, responses around this theme were inconsistent. For some, communication of uncertainty, for example, through discussion about ‘what-ifs’ or unlikely but ‘scary’ diagnoses, was worrying.

**Table 4:** illustrative quotations regarding worry versus reassurance

Responses from participants who preferred the high communicated diagnostic uncertainty vignette	Responses from participants who preferred the low communicated diagnostic uncertainty vignette
‘I would prefer to be told more, rather than less and to be given detailed reasons why a test was/wasn’t being done. I would only go and research it all myself anyway, so if I did this, and recognised things that the doctor had told me, *I would feel more reassured* that not only had I been treated correctly, but that I had been treated as an intelligent adult’. ‘I felt that the communication in [the high communicated uncertainty video] was more complete, and more comprehensively conveyed that a very thorough investigation had been made, the conclusions and the reasons for them. *I felt more reassured*…that there was probably no serious cause to the headache’. ‘[The high communicated uncertainty video] with more information about the context of the condition *was more reassuring* as you knew, rather than just suspected, that you had been screened for the really scary things’. ‘[The low communicated uncertainty video] left me wondering what other conditions might have caused the headache, and given the opportunity, I would have asked the question. The [high communicated uncertainty video] considered the alternatives, which is more satisfactory and ultimately, *more reassuring*’. ‘With [the low communicated uncertainty video] the patient could go home and *worry *about a condition not specifically mentioned by the doctor for example, cancer’. ‘“[The high communicated uncertainty video] mentioned illnesses that had been ruled out. [The low communicated uncertainty video] did not. If it was my experience, *I would be overthinking *about what tests had actually been done (eg, did he check for cancer) because he never specified (instead just saying “sinister illnesses”). I like to have as much of the key information as I can, especially about personal health’.	‘In [the high communicated uncertainty video] the doctor flip-flopped between “we don’t think there’s anything worrying” and “there might be/have been something worrying”. All the extra information created *extra confusion and worry* which was unnecessary. The [low communicated uncertainty video] made it clear that the doctor didn’t think there was anything to be worried about, but *reassured* that if I felt it necessary I could come back again. I would have gone home confident that I had been treated appropriately’. ‘The doctor on the [high communicated uncertainty video] gave the patient too many “what ifs” after saying everything was okay which would cause the patient to be *more concerned* about their health’. ‘I feel the [high communicated uncertainty video] gives too much information and would be *scary and cause me to overthink the issue’*. ‘I’m more concerned about whether my results are normal, and trust that any abnormalities would be spotted and communicated to me. Hearing “what could have been” *isn’t reassuring*’. ‘I don’t think you needed to mention cancer and in [the high communicated uncertainty video] it *made me feel a bit nervous* that they said it is unlikely to be cancer but they didn’t say it definitely wasn’t’. ‘The [high communicated uncertainty video) included information I did not need, may not understand, *created anxiety* and expressed uncertainty about the quality of the investigation, diagnosis and treatment to date’.

*I feel the [high communicated uncertainty video] gives too much information and would be scary and cause me to overthink the issue*. – P46

A small number of participants explicitly stated that even though they found the information in the high communicated uncertainty video more worrying, they still preferred it. These participants acknowledged that receiving more information about diagnostic uncertainty might be anxiety-inducing, but felt that the benefits of a fuller explanation outweighed this worry.

*I would prefer to be aware of all of the information—what the doctor is sure of, and what can’t be ruled out…Although, of course, [this] made me feel more worried…I would always rather know the full truth*. – P39

*[A]lthough knowing more about what the ‘worst case scenario’ might be (and that’s a bit scary and more worrying), I’d rather have the full picture*. – P19

In contrast, others described how the fuller explanation of uncertainties provided reassurance: communication of details about the differentials which were being considered helped to relieve worry, by making participants feel as though their concerns had been more thoroughly considered. Some noted that information can easily by looked up online, and they would rather be informed about uncertainties directly rather than coming across them themselves at home.

*I think that in the days of Google—many people could have an idea of what the symptoms could be. I would want it explicitly acknowledged that the Dr has considered…cancer for instance. It makes you feel like you are taken seriously*. – P4

In a similar vein, other participants stated that the omission of information in the low communicated uncertainty vignette was itself worrying. These participants explained that *not* being given all the information about possible diagnoses might cause anxiety.

#### Impact of communication on perceptions of the doctor

The impact (positive or negative) that the communication had on participant perceptions of the doctor was referenced. Acknowledging diagnostic uncertainty was sometimes perceived positively, taken as an indication that the doctor was honest and trustworthy:

*I preferred the fact that the doctor in [the high communicated uncertainty video] used words to reflect a (small) level of uncertainty like ‘likely’ or ‘unlikely’. That made me feel like he was being more honest*. – P15

*By the doctor explaining the differential diagnoses…this will illicit trust from the patient*. – P7

A small number of participants described how the communication of diagnostic uncertainty had a negative impact on their perception of the doctor, as it gave an impression of lack of confidence.

*I didn’t like that there was the offer to do the Lumbar test on second return, as it added an element of lack of confidence on the Drs part*. – P58

#### Impact of communication on health behaviours

Some participants mentioned the impact that the communication of uncertainty might have on future health behaviours. Some participants who preferred the high communicated uncertainty vignette suggested that it might make patients more likely to follow safety-netting advice. It is, however, important to note that some participants who preferred the low communicated uncertainty vignette also reported that they felt able to return after watching this video, suggesting that patients may be empowered to follow safety-netting advice based on factors other than the communication of diagnostic uncertainty.

*[The high communicated uncertainty video] would also make me more likely to look for help if I felt worse as I know there is uncertainty in the diagnosis*. – P15

*If symptoms don’t go away I am definitely coming back to ED. After the [low communicated uncertainty video] I would have wavered about coming back*.” – P19

A small number of participants describe how the high communicated uncertainty vignette might make them want further investigations.

*In [the high communicated uncertainty video]…having some big names thrown around like cancer made me more worried, and would be more insistent on getting tests, which probably are not needed at this point*. – P20

## Discussion

### Summary of empirical findings

This study indicates that, overall, patients are likely to prefer greater communication of diagnostic uncertainty, even though this may be more worrying—but this preference is not universal. Although quantitative data did not demonstrate significant differences in secondary outcomes such as trust, understanding, perception of doctor competence or reported likelihood of returning to seek medical advice, patients did refer to these topics in their free-text responses. The varied nature of the reasons given for their preferred vignette in the free-text responses may explain the few significant findings in the quantitative secondary outcomes: different participants sometimes gave directly opposing justifications for their preferences. There were no significant associations between demographic characteristics (eg, sex or educational level) and communication preferences.

The study examined two general medical presentations (headache and CoBH), with broad differential diagnoses. Although our findings are not necessarily generalisable to all other clinical scenarios, the principles discussed below (in particular the need to balance harms and benefits of information disclosure) are still widely relevant. Further research will be helpful in exploring diagnostic uncertainty communication in other kinds of clinical scenarios, for example where the symptoms are more indolent or where the investigations themselves are less conclusive (such as Parkinson’s disease or motor neuron disease).

Our data cannot alone provide definitive answers to the normative question of how doctors should communicate diagnostic uncertainty to their patients. The insights provided are, however, important in empirically grounding such discussions. If incorrect assumptions are made about what patients want to be told or what the effects of communicating diagnostic uncertainty to them are, we risk building ethical arguments founded on flawed conjectures, which may not reflect the realities of doctor-patient communication.

### Balancing harms and benefits of disclosure

Therapeutic privilege describes the right of the doctor to not disclose material information if there is a reasonable belief that it will result in serious harm to the patient.^4 54 55^ Ethical analyses of the therapeutic privilege typically consider how the potential harms of disclosure (eg, aversive patient responses to distressing information) must be balanced against the potential benefits (eg, the positive impact on patient autonomy).^56^ Our empirical data can help inform doctors in balancing the harms versus benefits of disclosing diagnostic uncertainty.

Perhaps the most commonly identified benefit of greater information disclosure is that it will promote patient autonomy. Autonomy is widely recognised as a central concept in bioethics, but various conceptualisations exist.^57 58^ Beauchamp and Childress define autonomy as the right to self-governance, to act in accordance with a self-chosen plan with intention, understanding and non-interference.^59^ Many bioethicists draw on this in employing a procedural definition (according to which an agent is autonomous with respect to an action if it is (i) voluntary, (ii) intentional and (iii) informed).^59^,^61^ Such procedural accounts contrast with Kantian conceptions of autonomy, in which the focus is not on autonomous *decisions* but on autonomous *persons*—individuals whose actions are determined by impartial and abstract principles of reason.^61^ Stirrat and Gill, building on O’Neill’s work, put forward a version of patient autonomy which requires ‘the provision of sufficient and understandable information and space for patients, who have the capacity to make a settled choice about medical interventions on themselves, to do so responsibly in a manner considerate to others’.^62^

Common to many of these conceptions of autonomy is the idea that it can be limited by inadequate provision of information.^63^ Ethical discussions around disclosure in the doctor-patient relationship commonly link enhanced information provision to greater patient autonomy: by being more ‘fully informed’, patients are better able to make decisions regarding their health. Of course, the provision of more information does *not* always result in greater autonomy—patients must be able to understand the information provided, so the provision of information which is overwhelming or overly technical may restrict, rather than promote, autonomy. Indeed, Rees has argued that in some in rare cases withholding information might actually promote a patient’s autonomy.^64^

In our study, the most common justification for preferring the high communicated uncertainty vignette was an appreciation of the additional information provided. The majority of participants valued greater discussion, including about uncertainties in investigations and alternate diagnoses being considered; this often facilitated a deeper understanding of their medical situation, which was construed positively. Proponents of doctor-patient communication often emphasise shared understanding as important: to promote patient autonomy and foster more informed decision-making, but also to help build trust in the doctor-patient relationship and enhance patient emotional well-being.^65^

Some of our participants explicitly linked having an increased understanding to the ability to make informed decisions, for example, seeking further medical attention in the event of worsening or non-resolving symptoms. In our qualitative data, a small number of participants stated that communicating diagnostic uncertainty might be helpful in facilitating appropriate reattendance; there was no significant difference in this outcome in the quantitative data. This discrepancy may be because only a small number of patients feel this way: although communicating uncertainty may influence some patients’ behaviours, others may be likely to follow safety-netting advice regardless. Another consideration is whether there is a significant a difference between participants’ reported and actual behaviours. Most participants in our study indicated that they would be very likely to return, suggesting a ceiling effect. Economic, logistical and emotional barriers to reattending hospital exist in real life, which our vignette methodology was unable to capture. As such, further research into real clinical practice would be helpful, examining whether communicating diagnostic uncertainty influences actual patient behaviours (as opposed to patient self-report of hypothetical behaviours).

It is also important to consider the potential benefits of shared understanding beyond patient agency. In our study, many participants did *not* link their understanding to the ability to make better decisions: they simply stated they preferred having more information to better understand their medical situation. Providing information about diagnostic uncertainty may thus be of value in facilitating shared understanding even if patients do not themselves want to make decisions about treatment or investigations. This is in keeping with research demonstrating that patients often have a high desire for information concerning their care, even if they have a lesser desire to be actively involved in decision-making.^66^,^68^ In this context, the importance of understanding patient preferences for both information provision and involvement in decision-making has been emphasised: as Murtagh and Thorns summarise, ‘[e]stablishing preferences enables us to show respect for patient autonomy in a manner that is sensitive and timely for that patient’.^69^

An oft-discussed potential harm of disclosing diagnostic uncertainty is that it will be distressing for patients. Our study validates some concerns raised by doctors in previous research^24 70^ that communicating diagnostic uncertainty may worry patients: the high communicated uncertainty vignettes were perceived as more worrying (although the effect size was small). Some of our participants explicitly stated that although they found the high communicated uncertainty video more worrying, they still preferred to receive this information. This aligns with a study of patients with endometriosis in which patients expressed a preference for the communication of diagnostic uncertainty, often conceptualising it as ‘the lesser of two evils’.^29^ Even though information about diagnostic uncertainty can be unsettling, patients may prefer it to ignorance on the matter. Politi et al have discussed the extent to which decisional dissatisfaction may be an unavoidable consequence of communicating uncertainty in scientific evidence to patients.^71^ This idea can be extended to the communication of diagnostic uncertainty: it might be that some anxiety is an inevitable consequence—a ‘necessary evil’—for some patients if doctors communicate diagnostic uncertainty information with the intention of promoting autonomy.

In summary, our data highlight that patients may prefer to receive more information about diagnostic uncertainty, even if this information is worrying. This has implications for how doctors should weigh up the potential harms and benefits of information disclosure in this context—many patients value the communication of diagnostic uncertainty in facilitating better understanding of their medical situation, despite the potential harm induced by the creation of worry. It is, however, important to acknowledge that the preference for greater diagnostic uncertainty communication was not universal. As we discuss in the next section, this presents a significant challenge for doctors.

### Communicating in the face of heterogeneous information preferences

We demonstrated variation in patient informational preferences—although most participants preferred greater communication of diagnostic uncertainty, a minority found the amount of information provided in the high communicated uncertainty videos unhelpful/unnecessary. This heterogeneity identified aligns with existing research examining doctor-patient communication more generally: studies have demonstrated variable patient informational desires in a range of clinical contexts (including in oncology,^1772^,^77^ end-of-life care^78^ and surgery^79^,^81^). This presents a challenge for clinicians, particularly in acute settings where there may not be a pre-existing doctor-patient relationship: how can doctors determine whether the specific patient in front of them will appreciate additional diagnostic uncertainty information?

One approach might be to use demographic characteristics to help guide communication. We did not find any significant associations between demographics and communication preference; this adds to mixed results in the existing body of literature exploring whether patient demographics can predict informational needs.^82^ Although some studies have found characteristics such as age,^72 73 83 84^ sex^73 85 86^ or educational level^78 84 87 88^ to be associated with informational preferences, as a review in oncology concluded: ‘demographics do not reliably predict individual informational preferences, and studies have found contradicting results’.^74^ Moreover, using demographic characteristics to predict information preferences raises its own ethical issues. Studies have demonstrated that black patients receive less information from their doctors compared with white patients^89^,^91^ and that clinician implicit race bias is associated with markers of poor communication.^92^ The use of patient characteristics such as ethnicity or perceived educational level to determine how much information to provide risks perpetuating such implicit biases and contributing to health inequalities.

An alternative might be to explore patient attitudes towards risk/uncertainty to help guide communication (eg, using the IUS-12, a 12-item instrument measuring reactions to uncertainty/ambiguous situations).^43^ It is plausible that patients with greater intolerance to uncertainty might be less likely to value explicit communication of diagnostic uncertainty. In our study, we did not find any significant associations between IUS-12 scores and primary or secondary outcomes. This suggests that exploring patients’ tolerance to uncertainty in general terms may not helpfully predict their information preferences or how they will respond to uncertainty communication. The logistical challenges presented by using such scale must also be considered—even if a scale was to be developed that accurately predicts patient desire for uncertainty information, it would also need to be practically feasible for use. Such logistical barriers might account for why some such scales have been developed,^67 68 93 94^ but are not widely used.

Finally, doctors could directly ask patients what they want to be told to help guide communication. Although this approach has some appeal, several problems arise in relying on patients to inform their doctors what information they would like to receive. First, it is difficult—arguably impossible—for patients who are ignorant about something to indicate whether they want to be told about it. For example, in the headache scenario, many patients may not have considered that a CT does not exclude a subarachnoid haemorrhage. They are therefore unlikely to ask for more information about this—but this is not to say they might not appreciate the information if provided. Second, relying on patients to guide information disclosure assumes that patients know, and are able to articulate, their own informational preferences.^95^ In practice, patients do not always voice their worries: a study in primary care found that patients commonly have unvoiced concerns regarding possible diagnoses.^96^ Relying on patients to articulate their informational preferences may perpetuate health inequalities: patients from minoritised groups are less likely to ask questions and tend to adopt a more passive role in the doctor-patient relationship.^89 91 97 98^ There is thus a risk that some patients will feel less empowered to ask for information and will be disadvantaged.

Ultimately, the heterogeneity of views identified in this study presents a challenge for doctors in determining how much diagnostic uncertainty information to communicate to the specific patient in front of them. There are the problems associated with trying to base communication on demographic characteristics, scales or even patients’ own expressed wishes—not least the risk of perpetuating existing biases and inequalities.

When deciding how to communicate diagnostic uncertainty, doctors should work with patients to elicit their informational preferences as far as is possible—we echo Nease and Brooks in their assertion that ‘[r]igid recommendations about how much information patients should receive and the degree to which patients should be involved in medical decision making may be inappropriate when applied to individual patients’. Yet it may not always be feasible to fully explore patient informational preferences in acute settings, and patients themselves may sometimes be unable to articulate such preferences. Pragmatically, we suggest that doctors could employ a utilitarian approach to address this challenge. If, as our study suggests, the majority of patients have a preference for greater disclosure of diagnostic uncertainty, then doctors should generally disclose this information (unless the patient has made it clear that they do not want this information)—as this will produce the best outcome for the majority of patients. When faced with a situation where it is not clear how much information a patient might want, we argue that the most ethical approach is to err on the side of greater disclosure of uncertainty information.

### Strengths and limitations

Unlike observational studies of real consultations, this study’s vignette methodology permitted communication behaviours to be isolated and manipulated, allowing inferences to be drawn about causal relationships between diagnostic uncertainty communication and various outcomes. A strength of the crossover design was that participants were able to watch both the high and low communicated uncertainty vignettes and directly compare them, indicating their preference. The inclusion of a free text section on the questionnaire permitted the collection of qualitative data, which was helpful in illustrating and interpreting the quantitative findings.

We chose to focus on two clinical scenarios, both of which are commonly encountered in general medicine. The fact that there was little difference between the responses given by participants who watched the headache vignettes versus the CoBH vignettes suggests some of our findings may be generalisable to other clinical scenarios, but further research is needed to explore this possibility.

An inherent weakness of vignette methodology relates to the degree to which participants respond to vignettes as they would a real consultation. Previous research has suggested that analogue patients can be included as proxies for clinical patients in studies on communication,^99^ and our pilot-testing results demonstrated that the vignettes had adequate internal and external validity. The vignettes were developed using established guidelines,^42^ with input from experts and laypeople in their development to make them as realistic as possible. Additionally, we used data collected from a previous study to develop the scripts, meaning that they reflected what real doctors would say in communicating diagnostic uncertainty. Thus, although responses to the vignettes may not be wholly equivalent to real consultations, they were as realistic as possible, and it is likely that our participants were able to respond to them in way which reasonably reflects real patient reactions.

As part of the pilot-testing process, we tested immersion and perceived realism of the vignettes—the final videos used in the main study performed well.^41^ We did not, however, test this in the main study (in part due to concerns about overburdening participants with multiple questionnaires); it is something we would consider doing in future vignette studies.

The nature of the study design precluded any conversation between participants and the doctor—the vignettes all depicted a doctor monologue to camera. Real consultations typically involve at least some degree of conversation, with patients asking questions and discussing information with their doctor. This study was unable to examine the effect of communicating diagnostic uncertainty in the context of a dialogue between patient and doctor. Although attempts were made to ensure that the sample was as diverse as possible, we acknowledge that our sample had a higher level of university education than the general population, likely a result of self-selection of individuals who chose to participate. We did not, however, find any significant difference in the primary outcome (preferred vignette) when we compared those with a university degree or higher and those without.

## Conclusion

Our study shows an overall patient preference for greater communication of diagnostic uncertainty, notwithstanding the potential for higher communicated uncertainty to be marginally more worrying. This provides some empirical support for recommendations to openly communicate diagnostic uncertainty to patients: although diagnostic uncertainty information might worry patients, they may still want to understand it.

We note that patient preferences are not uniform, and it can be difficult for clinicians to determine what an individual patients might want to be told, particularly in the absence of an existing relationship – but we caution against using demographic data to make assumptions about informational preferences, as this may create or exacerbate health communication inequalities.

Overall, we suggest that clinicians should err towards greater communication of diagnostic uncertainty to their patients. If healthcare professionals elect not to engage in dialogue with patients about diagnostic uncertainty (based on benign paternalistic ideas about avoiding patient worry) or do so variably (based on unevidenced assumptions about patient information preferences), they risk depriving patients of information they may value.

## Supplementary material

10.1136/jme-2024-109932 online supplemental file 1

## Data Availability

Data are available upon reasonable request.

## Footnotes

Note that in an AB|BA design, a carry-over effect cannot be distinguished mathematically from an interaction effect (between treatment (A or B) and period (first or second)), or a sequence effect (AB or BA).
