# Supplementary material for: To what extent should doctors communicate diagnostic uncertainty with their patients? An empirical ethics vignette study
Source: J Med Ethics. 2025 Feb 26;51(11):e109932. doi: 10.1136/jme-2024-109932 (PMC12573412; doi:10.1136/jme-2024-109932)
Supplement: online supplemental file 1 [file jme-51-11-s001.pdf]

Appendix A: Copy of Questionnaires

1 Post-video questionnaires

After watching each vignette, participants will complete the following questionnaire:

Please answer the following questions, imagining that you are the patient in the video that you have just watched.

For each statement below, please rate your response on a 0-10 scale:

1. To what extent did the information discussed in this consultation make you feel **worried**:

0	1	2	3	4	5	6	7	8	9	10
Not at all worried				Moderately worried						Extremely worried

2. Based on this consultation, how would you rate the **competence** of the doctor?

0	1	2	3	4	5	6	7	8	9	10
Not at all competent				Moderately competent						Extremely competent

3. To what extent would you **trust** this doctor to manage your medical problems?

0	1	2	3	4	5	6	7	8	9	10
I would not trust them at all			I would trust them moderately							I would trust them entirely

4. How **satisfied** were you overall with the consultation?

	0	1	2	3	4	5	6	7	8	9	10
Not at all satisfied					Moderately satisfied						Extremely satisfied

5. How did you feel about the **amount of information** provided by the doctor during this consultation?

	0	1	2	3	4	5	6	7	8	9	10
Not enough information						Just right					Too much information

6. To what extent did you **understand** the information the doctor discussed with you?

	0	1	2	3	4	5	6	7	8	9	10
I understood very little				I understood a moderate amount							I understood all of it

7. Based on what the doctor told you in this consultation, to what extent would **you want to have further tests** to investigate the cause of your symptoms?

	0	1	2	3	4	5	6	7	8	9	10
I would not want any further tests at all				I am indifferent to having more tests							I would definitely want more tests

8. How **likely would you be to seek medical attention** if your symptoms did not improve or got worse?

	0	1	2	3	4	5	6	7	8	9	10
Not at all likely					Moderately likely						Extremely likely

After watching both vignettes and completing both questionnaires, participants will be asked a final question about vignette preference:

In answering the following questions, think about both the video clips that you have watched.

If it were you, which type of communication would you prefer from your doctor?

1. Video A
2. Video B

Please provide your reasons for this preference:

2 Demographics and baseline measures

Finally, we would like to learn a little more about you. We are asking for this information so we can ensure and report on the diversity of our sample, and to help us interpret our results.

(1) How old are you?
   a. 18 – 29
   b. 30 – 39
   c. 40 – 49
   d. 50 – 59
   e. 60 – 69
   f. 70 – 79
   g. 80 or over

(2) What is your sex? A question about gender identity will follow.
   a. Male
   b. Female

(3) Is your gender you identify the same the sex you were registered at birth?
   a. Yes
   b. No (please describe your gender identity)

(4) What is the highest level of education you have completed? [choose one only]
   a. Primary school
   b. Secondary school up to 16 years
   c. Higher or secondary or further education (A-levels, BTEC, etc.)
   d. College or university
   e. Post-graduate degree
   f. Prefer not to say

(5) In what region do you currently live?
   a. North East
   b. North West
   c. Yorkshire and the Humber
   d. East Midlands

e. West Midlands
f. East of England
g. London
h. South West
i. Wales
j. Scotland
k. Northern Ireland

(6) What is your ethnic group?

1. White
    a. English / Welsh / Scottish / Northern Irish / British
    b. Irish
    c. Gypsy or Irish Traveller
    d. Roma
    e. Any other White background
2. Mixed / Multiple ethnic groups
    f. White and Black Caribbean
    g. White and Black African
    h. White and Asian
    i. Any other Mixed / multiple ethnic background
3. Asian / Asian British
    j. Indian
    k. Pakistani
    l. Bangladeshi
    m. Chinese
    n. Any other Asian background
4. Black / African / Caribbean / Black British
    o. African
    p. Caribbean
    q. Any other Black / African / Caribbean background
5. Other
    a. Arab
    b. Any other ethnic group, please describe
6. Prefer not to say

(7) Previous healthcare experience:

a. Have you ever visited you doctor with problems with change in your bowel habits?
    i. Yes
    ii. No
    iii. Prefer not to say
b. Have you ever attended A&E with a severe headache?
    i. Yes
    ii. No
    iii. Prefer not to say

We have some questions about how you think about uncertainty in your life in general. There are no right or wrong answers. Try not to spend too long thinking about your responses, but choose the option that feels most suitable for you.

For each statement below, please select the answer that best corresponds to how much you agree or disagree with each statement.

Not all characteristic of me – A little characteristic of me – Somewhat characteristic of me – Very characteristic of me – Entirely characteristic of me

a. Unforeseen events upset me greatly.
b. It frustrates me not having all the information I need.
c. Uncertainty keeps me from living a full life.
d. One should always look ahead so as to avoid surprises.
e. A small unforeseen event can spoil everything, even with the best planning
f. When it's time to act, uncertainty paralyses me.
g. When I am uncertain I can't function very well.
h. I always want to know what the future has in store for me.
i. I can't stand being taken by surprise.
j. The smallest doubt can stop me from acting.
k. I should be able to organise everything in advance
l. I must get away from all uncertain situations.

3 Rationale for using single-item scales

In the existing literature, some studies have used multiple questionnaire items to examine a single outcome (for example, a study examining patient perception of physician competence used 4 questions, which were combined to give an overall score).[1] In contrast, other studies have used single items to measure outcomes of interest (examples include a study which assessed trust using 0-10 numeric rating scale,[2] and a study which assessed patient satisfaction using a single 1-10 scale[3]). Although multi-item scales are often preferred over single-item scales on account of their psychometric advantages,[4] they are not without their disadvantages. For example, they may be time-consuming and result in participant burden; in addition, high levels of redundancy in items can spuriously inflate internal consistency.[5-7]

Single-item scales have been used successfully,[8 9] including in the healthcare setting.[10] As Postmes et al note, "*research supports the intuitively plausible idea that short scales may reach quite acceptable levels of reliability if they assess homogeneous, theoretically deduced, and clearly defined concepts*".[9] Factors that support use of single-item measures include when the constructs of interest are concrete, when the sample sizes and effect sizes are small, and in exploratory research.[5 6 11] It has also been noted that single-items may also be useful when asking respondents to provide an overall evaluation of more complex phenomena: "*for example, a general question concerning subjects' overall satisfaction with their communities (although clearly multidimensional) may be more relevant than simply summing their expressed satisfaction with various facets of community*".[12]

Overall, although single-item scales have their weaknesses, we felt that on balance single-item measures were more appropriate for this study. This is because:

- We are interested in a range of variables, so using multi-item scales for each would result in an impractically long questionnaire;
- Our research is exploratory, and the effect sizes may well be small;
- The constructs we are interested in are relatively homogenous and clearly defined.

We note that single item measures have been successfully used in a number of healthcare communication vignette studies: to measure global rating of physician,[1] patient trust,[2 13] satisfaction,[3 13] likelihood of recommending doctor to a friend,[14] anxiety,[15] intentions to switch doctors,[16] and patient uncertainty.[17]

REFERENCES

1. Saha S, Beach MC. The impact of patient-centered communication on patients' decision making and evaluations of physicians: a randomized study using video vignettes. *Patient Educ Couns* 2011;84(3):386-92.
2. Mori M, Fujimori M, Hamano J, et al. Which Physicians' Behaviors on Death Pronouncement Affect Family-Perceived Physician Compassion? A Randomized, Scripted, Video-Vignette Study. *J Pain Symptom Manage* 2018;55(2):189-97. e4.
3. van Osch M, van Dulmen S, van Vliet L, et al. Specifying the effects of physician's communication on patients' outcomes: A randomised controlled trial. *Patient Educ Couns* 2017;100(8):1482-89.

4. Rattray J, Jones MC. Essential elements of questionnaire design and development. *J Clin Nurs* 2007;16(2):234-43.
5. Bergkvist L, Rossiter JR. The predictive validity of multiple-item versus single-item measures of the same constructs. *Journal of marketing research* 2007;44(2):175-84.
6. Gardner DG, Cummings LL, Dunham RB, et al. Single-item versus multiple-item measurement scales: An empirical comparison. *Educational and psychological measurement* 1998;58(6):898-915.
7. Martinez-Martin P. Composite rating scales. *Journal of the Neurological Sciences* 2010;289(1-2):7-11.
8. de Boer AG, van Lanschot JJ, Stalmeier PF, et al. Is a single-item visual analogue scale as valid, reliable and responsive as multi-item scales in measuring quality of life? *Quality of Life Research* 2004;13(2):311-20.
9. Postmes T, Haslam SA, Jans L. A single-item measure of social identification: Reliability, validity, and utility. *British journal of social psychology* 2013;52(4):597-617.
10. Sendlbeck M, Araujo EG, Schett G, et al. Psychometric properties of three single-item pain scales in patients with rheumatoid arthritis seen during routine clinical care: a comparative perspective on construct validity, reproducibility and internal responsiveness. *RMD open* 2015;1(1):e000140.
11. Diamantopoulos A, Sarstedt M, Fuchs C, et al. Guidelines for choosing between multi-item and single-item scales for construct measurement: a predictive validity perspective. *Journal of the Academy of Marketing Science* 2012;40(3):434-49.
12. Willits FK, Theodori GL, Luloff A. Another look at Likert scales. *Journal of Rural Social Sciences* 2016;31(3):6.
13. Fujimori M, Shirai Y, Asai M, et al. Effect of communication skills training program for oncologists based on patient preferences for communication when receiving bad news: a randomized controlled trial. *J Clin Oncol* 2014;32(20):2166-72.
14. Hillen M, De Haes H, Stalpers L, et al. How can communication by oncologists enhance patients' trust? An experimental study. *Annals of oncology* 2014;25(4):896-901.
15. van Osch M, Sep M, van Vliet LM, et al. Reducing patients’ anxiety and uncertainty, and improving recall in bad news consultations. *Health Psychol* 2014;33(11):1382.
16. Hannawa AF. Disclosing medical errors to patients: effects of nonverbal involvement. *Patient Educ Couns* 2014;94(3):310-13.
17. Mori M, Fujimori M, van Vliet LM, et al. Explicit prognostic disclosure to Asian women with breast cancer: A randomized, scripted video-vignette study (J-SUPPORT1601). *Cancer* 2019;125(19):3320-29.
